# Supplementary material for: Chromatin modifier Hmga2 promotes adult hematopoietic stem cell function and blood regeneration in stress conditions
Source: EMBO J. 2024 May 29;43(13):7. doi: 10.1038/s44318-024-00122-4 (PMC11217491; doi:10.1038/s44318-024-00122-4)
Supplement: Supplementary file 1 — Appendix [file 44318_2024_122_MOESM1_ESM.pdf]

Table of contents 1

Appendix Figure S1. Deletion of the *Hmga2* gene in *Hmga2* KO mice. 2

Appendix Figure S2. The phosphorylation levels of CK2 substrates in stress conditions 3

Appendix Figure S3. Expression levels of *HMGA2* in normal aged HSCs and MDS HSPCs 4

Appendix Figure S1

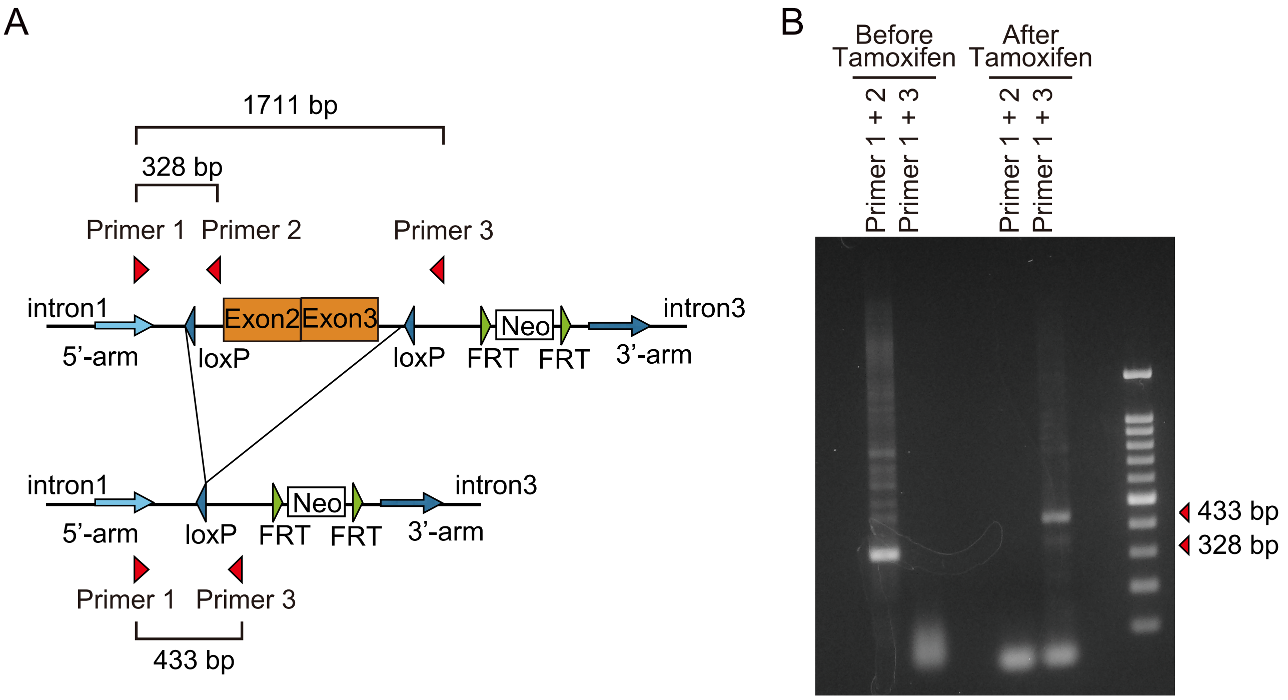

Appendix Figure S1.

A) Schematic diagram of the Hmga2 flox allele and the deleted allele with regions that were amplified by genomic PCR using primers (1, 2, and 3) indicated by arrows.

B) Representative image showing the Hmga2 deletion in Hmga2flox/flox;Cre-ERT2 mice after the tamoxifen injection. Arrowheads indicate the expected PCR products (328 bp by primers 1 and 2 and 433 bp by primers 1 and 3).

Appendix Figure S2

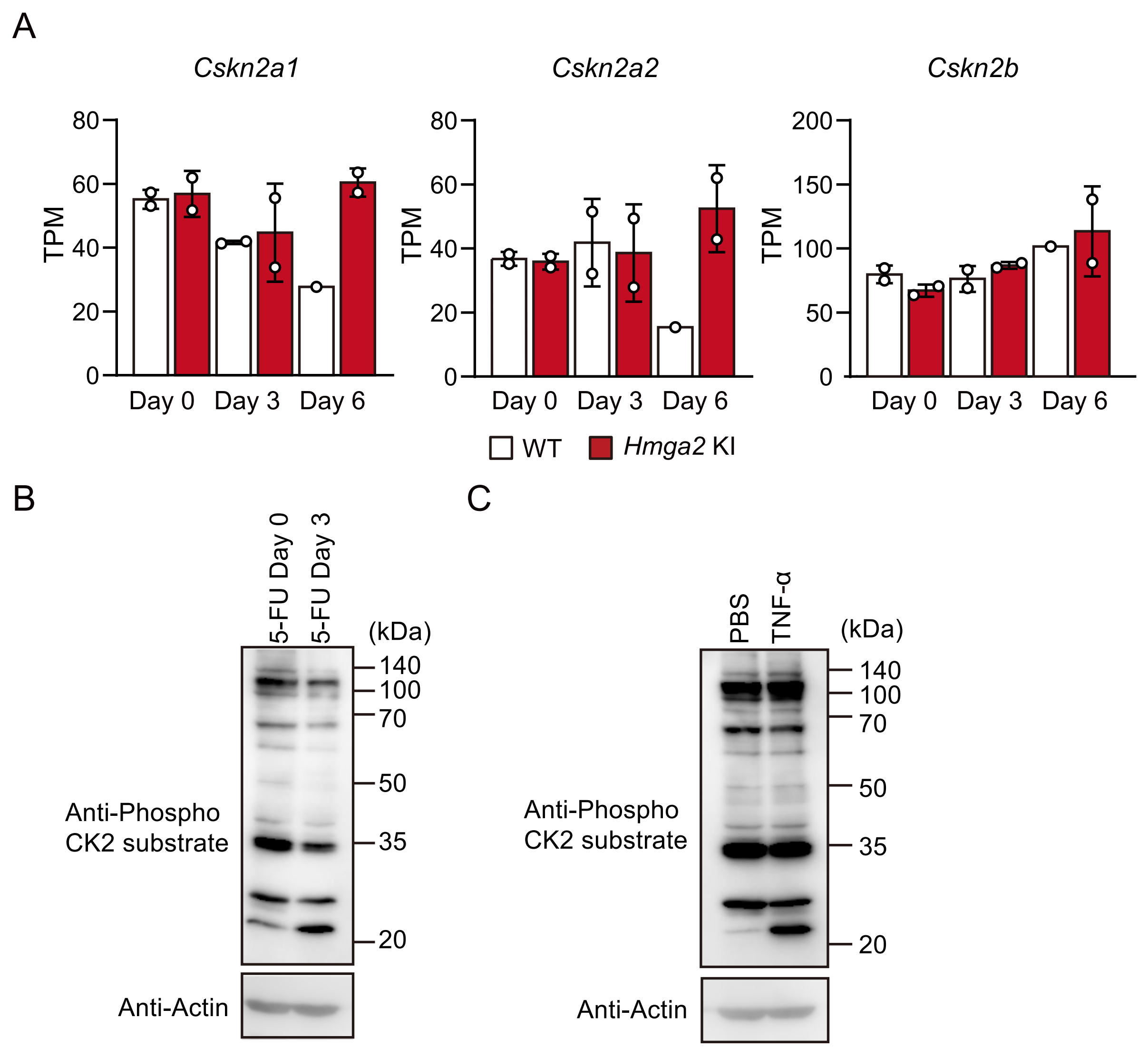

Appendix Figure S2.

A) Expression levels of CK2 family genes, such as Cskn2a1, Cskn2a2, and Cskn2b, in HSCs after the 5-FU injection examined by RamDA sequencing.

B) Western blotting of WT MNCs before and after the 5-FU injection using a phospho-CK2 substrate (CST, #8738) antibody. Actin was used as the loading control.

C) Western blotting of WT MNCs in a culture with or without 100 ng/ml TNF-α using a phospho-CK2 substrate (CST, #8738) antibody. Actin was used as the loading control.

Appendix Figure S3

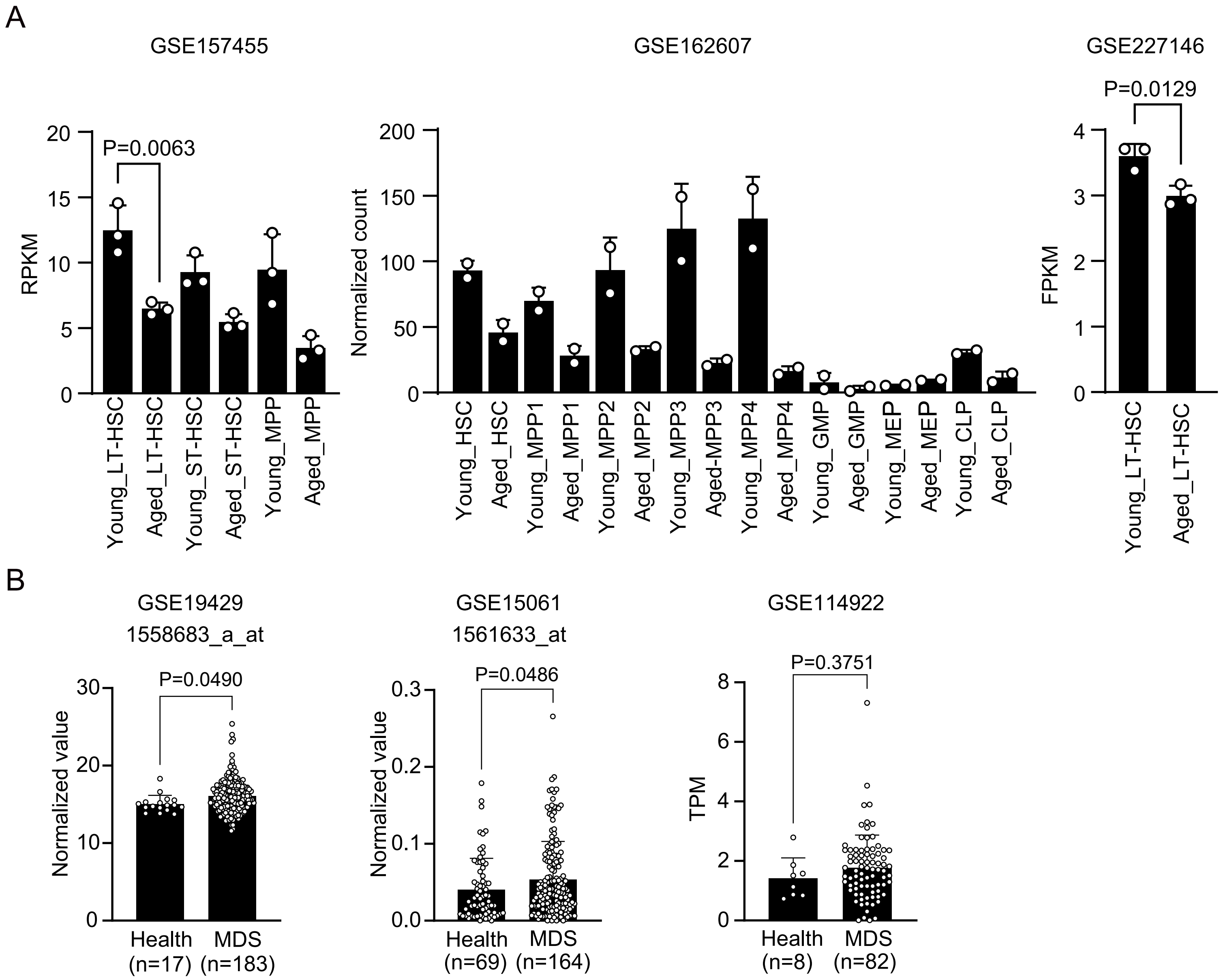

Appendix Figure S3.

A) Hmga2 mRNA expression level in HSCs and progenitor cells isolated from young and old WT mice (linked in GSE157455, GSE162607, and GSE227146).

B) HMGA2 mRNA expression levels in CD34+ HSPCs isolated from healthy individuals and patients with MDS (linked in GSE15061, GSE19429, and GSE114922).
