# Supplementary material for: Chromatin modifier Hmga2 promotes adult hematopoietic stem cell function and blood regeneration in stress conditions
Source: EMBO J. 2024 May 29;43(13):7. doi: 10.1038/s44318-024-00122-4 (PMC11217491; doi:10.1038/s44318-024-00122-4)
Supplement: Supplementary file 18 — Expanded View Figures [file 44318_2024_122_MOESM18_ESM.pdf]

Expanded View Figures

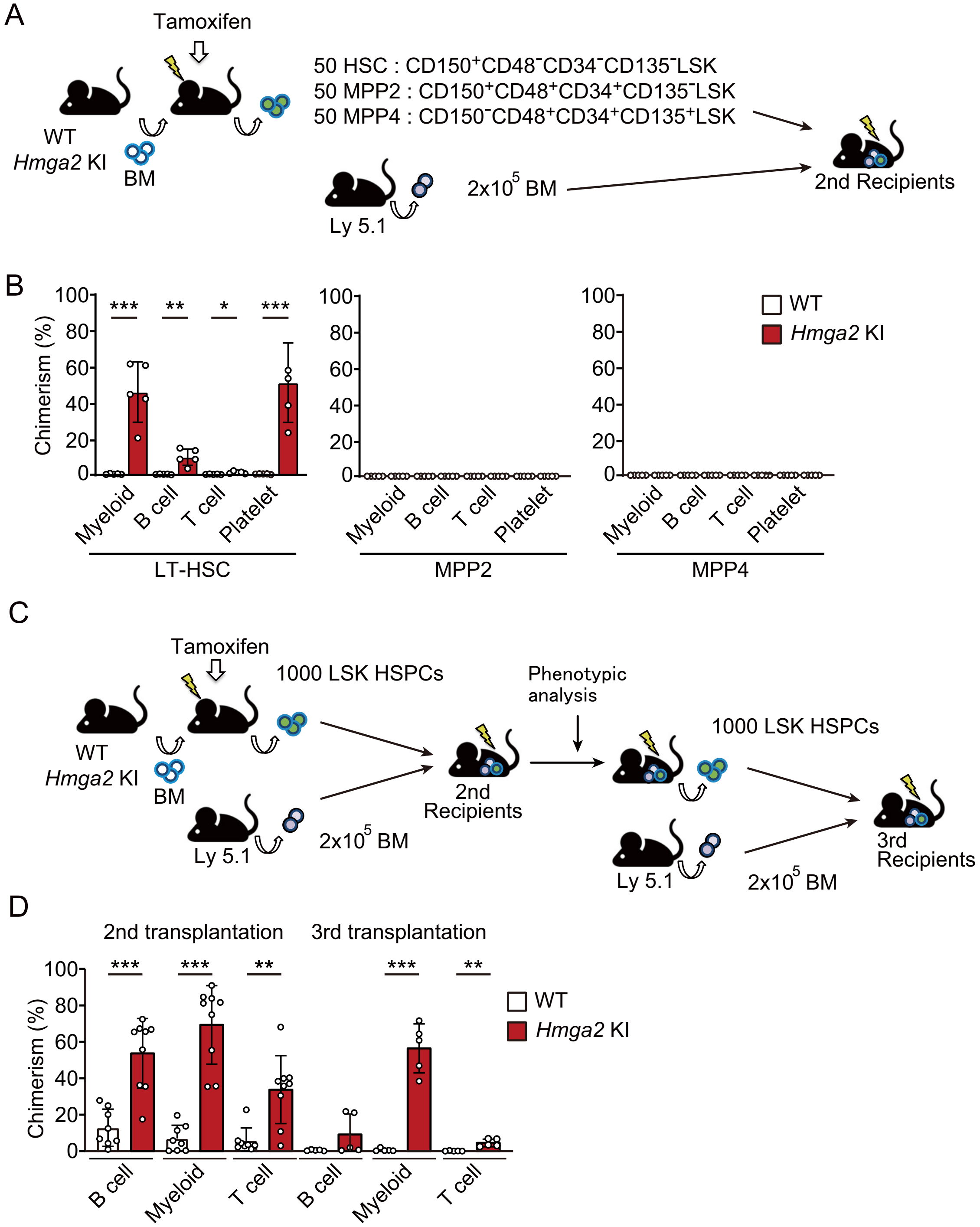

Figure EV1. Hmga2 overexpression enhanced the self-renewal of adult HSCs after transplantation.

(**A**) Schematic illustration of competitive transplantation showing 50 LT-HSCs, ST-HSCs, or MPP cells (CD45.2$^+$), which were isolated from *Rosa26-YFP* KI or *Hmga2* KI BM cell-transplanted mice 4 months after the tamoxifen treatment, and 2 × 10^5 WT BM cells (CD45.1$^+$) were transplanted into lethally-irradiated recipient mice (CD45.1$^+$). (**B**) Percentages of CD45.2$^+$ cells in Gr-1$^+$/CD11b$^+$ myeloid cells, B220$^+$ B cells, and CD4$^+$/CD8$^+$ T cells in PB at 4 months (n = 5). (**C**) Schematic illustration of competitive transplantation showing that 1000 LSK HSPCs (CD45.2$^+$), which were isolated from *Rosa26-YFP* KI or *Hmga2* KI BM cell-transplanted mice 4 months after the tamoxifen treatment, and 2 × 10^5 WT BM cells (CD45.1$^+$) were transplanted into lethally-irradiated recipient mice (CD45.1$^+$). (**D**) Percentages of CD45.2$^+$ cells in Gr-1$^+$/CD11b$^+$ myeloid cells, B220$^+$ B cells, and CD4$^+$/CD8$^+$ T cells in PB 4 months after secondary and tertiary transplantations (n = 5–9). Data information: In panel (**B**, **D**), bars show the mean ± SD, *$p < 0.05$, **$p < 0.01$, and ***$p < 0.001$. *P*-values were calculated by the Student's *t*-test. *N* means the number of mice. Data are representative of two independent experiments.

© The Author(s)

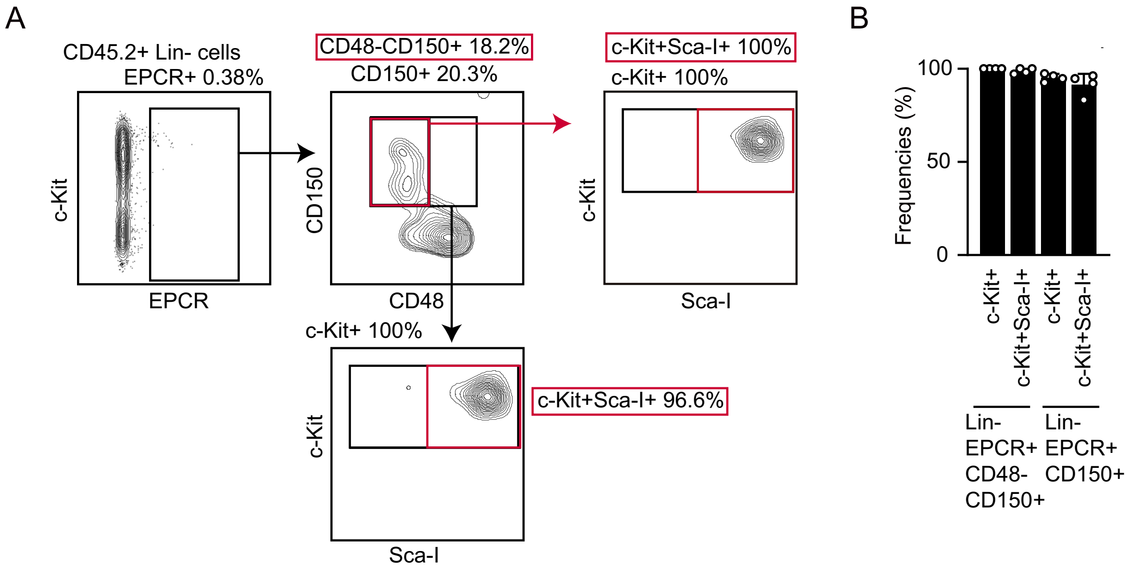

Figure EV2. Phenotypic definition of HSCs using EPCR.

(**A**) Gating strategy of the definition of HSCs and representative flow cytometry plots. (**B**) Percentages of Kit^+ or $Kit^+Sca\text{-}1^+$ among $Lin^-EPCR^+CD48^-CD150^+$ HSCs and $Lin^-EPCR^+CD150^+$ cells (*n* = 4; *n* means the number of mice). Bars show the mean ± SD.

 © The Author(s)

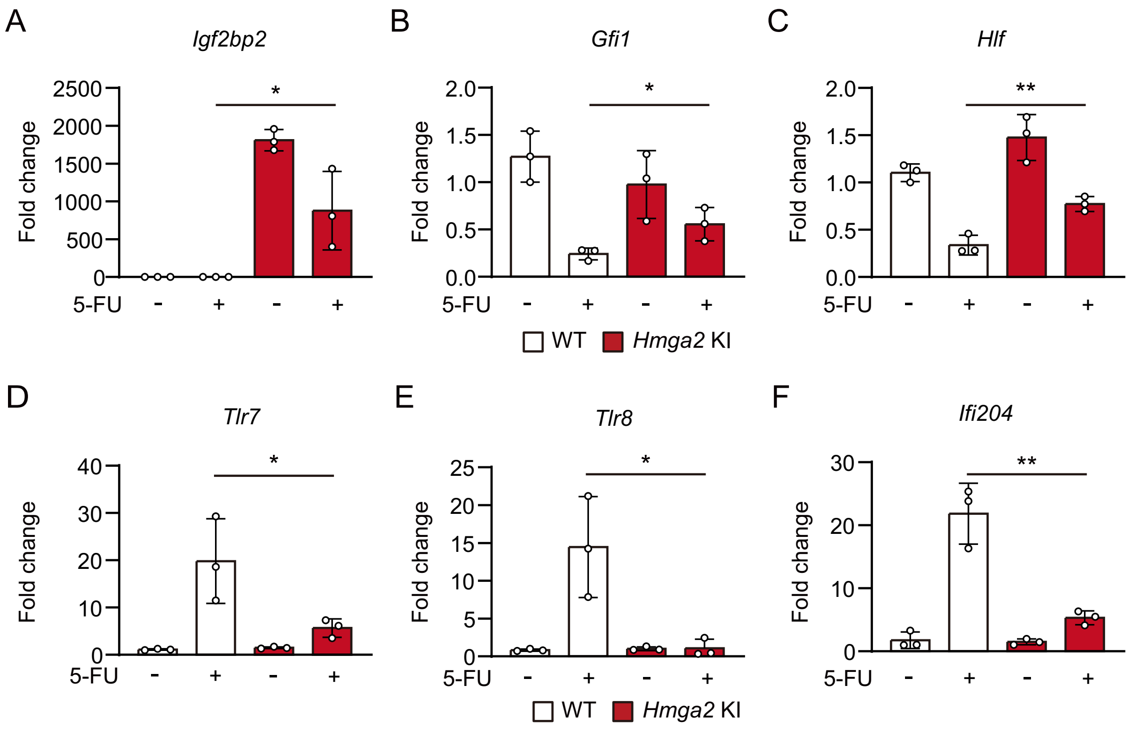

Figure EV3. Changes in gene expression in HSCs after the 5-FU injection examined by Q-RT-PCR.

(**A–F**) Gene expression levels of *Igf2bp2*, *Gfi1*, *Hlf*, *Tlr7*, *Tlr8*, and *Ifi204* in WT and *Hmga2* KI HSCs before and 3 days after the 5-FU injection examined by Q-RT-PCR ($n = 3$). Bars show the mean ± SD, $*p < 0.05$ and $**p < 0.01$. *P*-values were calculated by the Student's *t*-test. *N* means the number of samples.

© The Author(s)

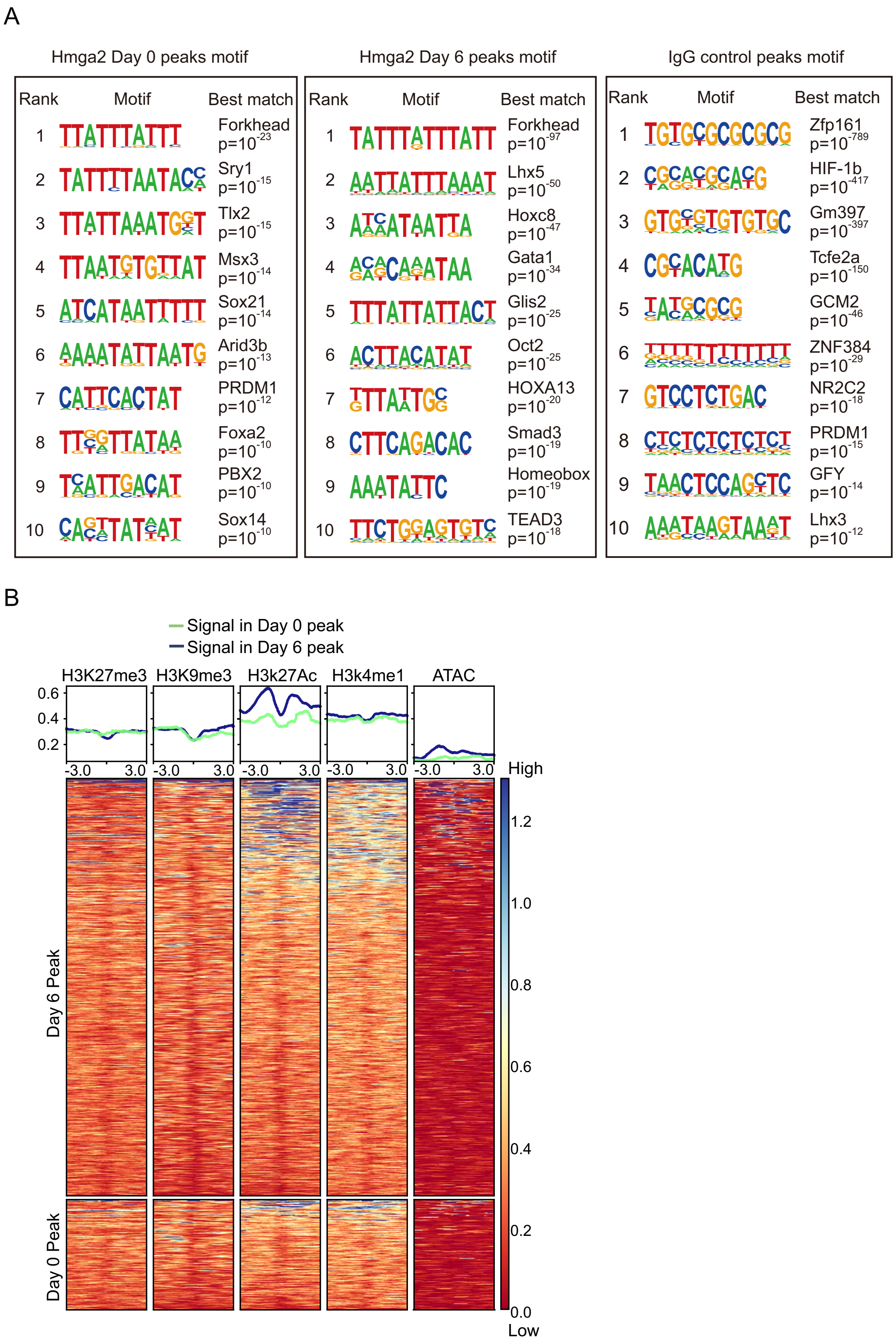
A
Hmga2 Day 0 peaks motif
Hmga2 Day 6 peaks motif
IgG control peaks motif
Rank
Motif
Best match
Forkhead $p=10^{-23}$
Sry1 $p=10^{-15}$
Tlx2 $p=10^{-15}$
Msx3 $p=10^{-14}$
Sox21 $p=10^{-14}$
Arid3b $p=10^{-13}$
PRDM1 $p=10^{-12}$
Foxa2 $p=10^{-10}$
PBX2 $p=10^{-10}$
Sox14 $p=10^{-10}$
Forkhead $p=10^{-97}$
Lhx5 $p=10^{-50}$
Hoxc8 $p=10^{-47}$
Gata1 $p=10^{-34}$
Glis2 $p=10^{-25}$
Oct2 $p=10^{-25}$
HOXA13 $p=10^{-20}$
Smad3 $p=10^{-19}$
Homeobox $p=10^{-19}$
TEAD3 $p=10^{-18}$
Zfp161 $p=10^{-789}$
HIF-1b $p=10^{-417}$
Gm397 $p=10^{-397}$
Tcfe2a $p=10^{-150}$
GCM2 $p=10^{-46}$
ZNF384 $p=10^{-29}$
NR2C2 $p=10^{-18}$
PRDM1 $p=10^{-15}$
GFY $p=10^{-14}$
Lhx3 $p=10^{-12}$
B
Signal in Day 0 peak
Signal in Day 6 peak
H3K27me3
H3K9me3
H3k27Ac
H3k4me1
ATAC
Day 6 Peak
Day 0 Peak
High
Low

 © The Author(s)

Figure EV4. Hmga2 bound to AT-rich and non-regulatory regions in chromatin.

(**A**) Motif enrichment analyses of Hmga2-ChIP-seq in HSPCs isolated from *HA*-tagged-*Hmga2* KI mice before and 6 days after the injection of 5-FU. Pre-immune IgG was used as a negative control. *P*-values were calculated by using gene ontology functions of HOMER software. (**B**) Heatmaps showing the levels of described histone modifications, such as H3K4me1, H3K9me3, H3K27ac and H3K27me3, and ATAC peaks identified in HSCs (linked in GSE119198, GSE60103, and E-MTAB-11865) in Hmga2-binding regions (Hmga2-binding site ± 3.0 kb) in HSPCs before and after the injection of 5FU.

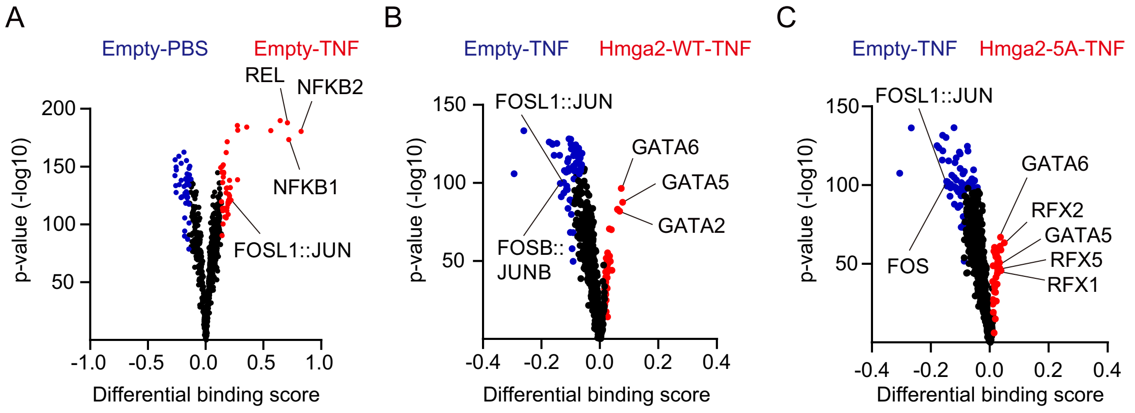

Figure EV5. Motif analysis of the open-chromatin region in each comparison.

(**A–C**) Volcano plots showing the differential binding activity of TFs between the indicated HSCs. Differentially binding score, transcription factor, and *p*-values were calculated by using TOBIAS software.

 © The Author(s)
